# Mental disorders and socioeconomic outcomes in women with cervical cancer, and their children and co-parents

**DOI:** 10.1093/jnci/djaf129

**Published:** 2025-06-10

**Authors:** Jiangrong Wang, Stina Salomonsson, Demet Sönmez, Sara Nordqvist Kleppe, Adina L Feldman, Marcus Sven Andersson, Goran Bencina, Fang Fang, Karin Sundström

**Affiliations:** Department of Clinical Science, Intervention and Technology, Center for Cervical Cancer Elimination, Karolinska Institutet, Stockholm, Sweden; Value & Implementation Outcomes Research, MSD, Stockholm, Sweden; Value & Implementation Outcomes Research, MSD, Stockholm, Sweden; Department of Clinical Science, Intervention and Technology, Center for Cervical Cancer Elimination, Karolinska Institutet, Stockholm, Sweden; Department of Clinical Science, Intervention and Technology, Center for Cervical Cancer Elimination, Karolinska Institutet, Stockholm, Sweden; Medical Affairs, MSD, Stockholm, Sweden; Value & Implementation Outcomes Research, MSD, Madrid, Spain; Institute of Environmental Medicine, Karolinska Institutet, Stockholm, Sweden; Department of Clinical Science, Intervention and Technology, Center for Cervical Cancer Elimination, Karolinska Institutet, Stockholm, Sweden; Medical Diagnostics Karolinska, Karolinska University Hospital Huddinge, Stockholm, Sweden

## Abstract

**Background:**

Cervical cancer often affects women who are in the middle of life and may carry substantial mental and socioeconomic impact also on families. We performed a generation-spanning study to elucidate this burden.

**Methods:**

We used nationwide registers during 1991-2018 in Sweden to perform 2 matched cohort studies based on a source population of more than 5 million women. The individual sub-study included 6060 cases of cervical cancer diagnosed during 2006-2018 and 5 population comparators individually matched to each case by age, birth year, and region (n = 30 300). The family sub-study included 9332 cases of cervical cancer diagnosed during 1991-2016 and 45 674 matched population comparators and all their children and co-parents.

**Results:**

We found an increased risk for mental disorders in cases compared with comparators, particularly during the first 2 years postdiagnosis (HR = 3.74, 95% CI = 3.45 to 4.06). Socioeconomic status changed negatively in cases after their diagnosis: a decreased income and increased need for financial aid appeared within 2 years, whereas unemployment escalated from 2 years after cancer diagnosis. We further found an increased risk of mental disorders in both children and co-parents of the cases, compared with the children and co-parents of the comparators. Furthermore, we observed negative socioeconomic trajectories in the co-parents and lower educational attainment in the children of the cases, especially if the case had died.

**Conclusions:**

Women with cervical cancer, and their close family members, display increased risk of negative mental health and socioeconomic outcomes after diagnosis. The lower educational attainment in children appears particularly worrying.

## Introduction

Patients diagnosed with cancer are at an increased risk of developing psychiatric disorders^1^ and about one-third experience depression or adjustment disorders after the diagnostic event.^2^ Cancer patients diagnosed with a stress-related mental disorder further display an increased risk for mortality compared with cancer patients who do not develop such disorders.^3^ These findings have been made in cancer populations in general, whereas less is known about female-specific cancers and particularly cervical cancer. We recently found a higher overall mortality in cervical cancer patients who had a preexisting diagnosis of mental disorder compared with patients without such diagnosis.^4^

Globally, cervical cancer is the fourth leading cause of cancer mortality and also the fourth most common cancer diagnosed in women.^5^ Cervical cancer can be prevented through 3 pillars: HPV vaccination, cervical screening, and effective treatment of precancerous lesions, as defined by the World Health Organization.^6^ For the women who are diagnosed with manifest cancer, appropriate clinical care and adequate posttreatment psychosocial support can reduce the consequences of the disease in her and her family members; and such management may be informed by data on mental and socioeconomic trajectories after diagnosis.

Furthermore, support may also be needed for family members including (young) children of women with cancer, in particular should the mothers not survive their diagnosis. Said support could be guided by knowledge of potential mental and socioeconomic consequences among a patient’s family members. Yet, relatively little is understood about cancer impact in the partner and children of the female patient afflicted by cervical cancer. Such knowledge is challenging to generate due to the need for long-term data capable of accurately linking families and their health and financial data over time.

Studies on this key topic have used questionnaires and reported an increase in psychological distress and economic burden on the families of cervical cancer patients responsible for informal care in lower resource settings.^7-9^ These findings could be augmented by register-based studies in the population, which can cover additional aspects over time and generations.

We aimed to add to this knowledge base and further inform this issue through a nationwide study of all cases of cervical cancer occurring during 2006-2018 in Sweden with linkage to comprehensive follow-up data on mental health and socioeconomic outcomes in these women, as well as their children and co-parents.

## Methods

This was a noninterventional observational study using data from Swedish population-based registers. All Swedish citizens are at birth or immigration assigned a unique personal identification number (PIN) which allows for very high-fidelity linkage between registers.^10^ Linkage is carried out by a coordinating government agency (in this study, Statistics Sweden), after which the PIN is replaced with a random sequence number and thus only pseudonymized (coded) data are available to the researchers.

### Data sources

For this study, we linked 6 national register data sources: the Swedish Cancer Register, The National Patient Register (NPR), the Swedish Prescribed Drug Register, the Swedish Cause of Death Register, the Multi-Generation Register, and the socioeconomic LISA database. All these are described in detail in Supplementary Methods.

### Study population

We performed 2 matched cohort sub-studies to examine the association of cervical cancer with the risk of mental disorders and socioeconomic changes among the individual women (individual sub-study) and their families (family sub-study).

In the individual sub-study, we defined the source population as all 5 614 005 women who were born between 1909 and 1998 and resident in Sweden at any time during 2006-2018. We included as cases all women with a cervical cancer diagnosis during 2006-2018 and matched 5 randomly sampled comparators per case from the source population by using the method of incidence density sampling. Comparators were individually matched to the case on calendar year of birth and were required to be free of cervical cancer at the diagnosis date of the case (ie, the index date). This resulted in a 1:5 age- and calendar-year-matched cohort of 6060 cases and 30 300 comparators.

We followed both the cases and comparators from the index date until the occurrence of a specific event of outcome, death, emigration, or 31st of December 2018, whichever came first. The analysis of mental disorders covered a study period of 2006-2018, as the Prescribed Drug Register used to identify mental disorders based on dispensations of prescribed psychiatric medications alone was established in July 2005.

In the family sub-study, we defined the source population of proband women as all 6 326 255 women who were born between 1894 and 1995 and resident in Sweden at any time during 1991-2016. We restricted the study period to 2016, instead of 2018 as in the individual sub-study, to allow at least a 5-year follow-up for the family members of the probands. We included as proband cases all women who were diagnosed with cervical cancer during 1991-2016 (*n* = 11 602) and matched 5 randomly sampled comparators per case from the source population, as in the individual sub-study (*n* = 58 010). Through linkage to the Multi-Generation Register, we further identified the biological and adoptive children born before the index (matching) date of the cases and comparators, as well as the legal co-parents of these children (legal co-parenting used as a proxy for identifying partners of the proband cases and comparators). All included co-parents thus had joint children with the proband woman.

This resulted in a matched cohort study of 9311 proband case and 45 590 comparators, 20 435 children of cases and 98 600 children of comparators, and 9272 co-parents of cases and 43 406 co-parents of comparators. In the family sub-study, the individual children and co-parents of the proband cases and comparators were followed from the index date, until occurrence of a specific event of outcome, death, emigration, or 31st of December 2021, whichever came first. Similar to the individual sub-study, the analysis of mental disorders covered the study period of 2006-2018, whereas the analysis of socioeconomic changes covered the entire study period of 1991-2021.

### Definitions of exposure and outcomes

The exposure was a diagnosis of invasive cervical cancer, that is, identified through ICD-7 code 171. ICD-7 was used consistently by the Swedish Cancer Registry during the duration of the study period; ICD-9 and ICD-10 were translated where needed to ICD-7, for harmonization purposes.

As the outcome, we defined mental disorder overall as a specialist diagnosis of any kind (ICD-10: F10-99), and further divided into substance abuse (ICD-10: F10-19 excluding F10 and F17), alcohol- and tobacco-related disorders (ICD-10: F10 and F17), psychotic disorder (ICD10: F20-29), depression (ICD-10: F32-33), anxiety (ICD-10: F40-41), and stress-related disorder (ICD-10: F43), according to the NPR (both inpatient and outpatient specialist care diagnoses included, see Table S1). To complement diagnoses made in specialist care, we added prescribed use of psychiatric medications as a proxy for mental disorders diagnosed in primary care, using Anatomical Therapeutic Chemical (ATC) codes N05A, N05B, N05C, and N06A for, antipsychotics, anxiolytics, hypnotics and sedatives, and antidepressants, according to the Prescribed Drug Register. Thus, a record of either any of these ICD diagnoses or a recorded dispensation of any of these ATC codes defined a case of “any mental disorder.”

Mental disorders were identified for each case and comparator, their children and co-parents, from the initiation of NPR and Prescribed Drug Register to the end of follow-up. A new diagnosis of a specific mental disorder after the index date was defined as the outcome of interest (the date of diagnosis as the outcome event date). Individuals with a pre-existing mental disorder before the index date were excluded from the analysis of this specific outcome. Individuals with a previous dispensed use of any of the above listed medications were similarly excluded from the analysis of such medicine use.

For our socioeconomic analysis, we first defined the baseline characteristics of income and employment 1 year before index date in both proband cases and comparators as well as their co-parents. We then focused on measuring incident negative outcomes in said characteristics after the index date. These negative outcomes included loss of employment, early retirement, receiving sickness benefit, and receiving family financial assistance, according to LISA (Supplementary Methods). A person categorized as having a negative outcome was defined as having had no such event in the year before the index date but having had such an event at some time following the index date. To structure these models, individual and family income was first categorized in quartiles in each calendar year according to the population distribution in the same 10-year age group: “low” if <25th percentile, “medium-low” if ≥25th and <50th percentile, “medium-high” if ≥50th and <75th percentile, and “high” if ≥75th percentile. A decrease in individual or family income was defined when the income level dropped from a higher quartile in the year before the index date to a lower quartile sometime after the index date. Thus, for example, participants who started at the lowest level of income and remained there throughout follow-up were not considered as having a negative outcome in the model. However, if they began also receiving family financial support during follow-up, they would be categorized as having that outcome.

Finally, we assessed educational attainment of the children of the proband cases and comparators, respectively, who were 18 or younger at the index date and 19 or older at the end of follow-up. Highest recorded level of education was defined in 7 categories in LISA: (1) primary school level education shorter than 9 years, (2) primary school level education of the full 9 years, (3) upper secondary school/high school level education for 2 years, (4) upper secondary school/high school level education for 3 years, (5) college/university/special professional training level education for shorter than 3 years, (6) college/university/special professional training level education of 3 years or longer (excluding postgraduate education), and (7) postgraduate education.

### Statistical analyses

Descriptive statistics were obtained using crude counts, proportions, and incidence rate calculations. χ^2^ test of differences by groups was omitted due to the large sample size. In the individual sub-study, we used Cox models stratified by the matching strata to assess the association of cervical cancer with mental disorders and socioeconomic changes at 0-<2, 2-<5, or 5-<12 years after cervical cancer diagnosis, adjusted for individual level of education in the year before index date, and country of birth. We estimated adjusted hazard ratios (aHRs) with 95% confidence intervals (CIs) for the overall analysis as well as by whether the cancer in case was categorized as late stage (defined as FIGO stage II+).

In the family sub-study of children, we used stratified Cox models to assess the association between having a mother diagnosed with cervical cancer and mental disorders in the children at 0-<2, 2-<5 and 5-<12 years after the index date, adjusted for mother’s age at the index date, mother’s education level, and family income level in the year before index date. We further stratified the analyses by the mother’s vital status after the index date, as a time-varying covariate, as well as the mother’s age at the index date and gender of the children. When analyzing children’s educational attainment, conditional ordinal logistic regression models were used to assess the relative risk of attaining a lower level of education in relation to having a mother with cervical cancer. These models adjusted for the children’s year of birth, age at index date, educational level of the mother, and family income level in the year before index date.

In the family sub-study of co-parents, we used stratified Cox models to assess the association between the proband’s cervical cancer status (case or comparator) and risk of mental disorders in the co-parent at 0-<2, 2-<5, and 5-<12 years after the index date. These models adjusted for age at index date and education level of the proband (case or comparator), and family income level in the year before index date.

In these models, we also stratified for the vital status of proband (case or comparator) as a time-varying covariate, to evaluate the potential effect modifier for severity of the disease. We further adjusted for the age, gender, education level, and country of birth of the co-parent.

All tests were 2-tailed and *P *< .05 was interpreted as statistically significant. Analyses were carried out using SAS v 11 and STATA v18.0.

### Ethical approval

Ethical approval was granted by the Swedish Ethical Review Authority (DNR 39628/2021), which determined that informed consent from the participants was not required.

## Results

### Characteristics of the study participants

Due to the matched design, case and comparators displayed an equal distribution of year of birth and age at index date. There were some differences regarding country of birth and level of education: cases were more likely Swedish born (84.6% vs 80.8%) and were more likely to have the lowest level of attained education, to be unmarried or divorced, and to have low family income than comparators (Table 1). Characteristics of co-parents and children are presented in Tables S2 and S3.

**Table 1.** Individual sub-study.

	Cases (%)	Comparators (%)
Total number of women	6060 (100%)	30 300 (100%)
Year of birth		
1909-1919	44 (0.7)	220 (0.7)
1920-1929	399 (6.6)	1995 (6.6)
1930-1939	610 (10.1)	3050 (10.1)
1940-1949	773 (12.8)	3865 (12.8)
1950-1959	799 (13.2)	3995 (13.2)
1960-1969	1057 (17.4)	5285 (17.4)
1970-1979	1420 (23.4)	7100 (23.4)
1980-1994	958 (15.8)	4790 (15.8)
Country of birth		
Sweden	5126 (84.6)	24 466 (80.8)
Other Nordic countries^a^	219 (3.6)	973 (3.2)
Other countries	715 (11.8)	4787 (15.8)
Missing	0 (0)	74 (0.2)
Age at cancer diagnosis (index/matching date)		
≤40	2089 (34.5)	10 445 (34.5)
41-50	1253 (20.7)	6265 (20.7)
51-60	812 (13.4)	4060 (13.4)
61-70	731 (12.1)	3655 (12.1)
71-80	675 (11.1)	3375 (11.1)
≥81	500 (8.3)	2500 (8.3)
Length of follow-up		
≤2 years	2012 (33.2)	5881 (19.4)
2-<5 years	1670 (27.6)	8491 (28.0)
5-10 years	1601 (26.4)	10 499 (34.7)
>10 years	777 (12.8)	5429 (17.9)
Education		
Low (less than 9 years)	1349 (22.3)	5955 (19.7)
Middle (9-12 years)	2700 (44.6)	12 265 (40.5)
High (13 years or more)	1888 (31.2)	11201 (37)
Missing	123 (2)	879 (2.9)
Marital status		
Unmarried	2089 (34.5)	9083 (30)
Married (registered partner^b^)	2132 (35.2)	13 718 (45.3)
Divorced	1220 (20.1)	4035 (13.3)
Widow	586 (9.7)	3019 (10)
Missing	33 (0.5)	445 (1.5)
Employment status		
Employed	5479 (90.4)	27 157 (89.6)
Unemployed	548 (9)	2698 (8.9)
Missing	33 (0.5)	445 (1.5)
Early retirement/Sickness benefit		
No	5582 (92.1)	27 823 (91.8)
Yes	445 (7.3)	2032 (6.7)
Missing	33 (0.5)	445 (1.5)
Family financial assistance		
No	3315 (94.5)	16 590 (94.6)
Yes	166 (4.7)	680 (3.9)
Missing	28 (0.8)	275 (1.6)
Individual income		
Lowest quartile	1021 (23.6)	5162 (23.8)
Second quartile	1115 (25.7)	5347 (24.7)
Third quartile	1096 (25.3)	5424 (25)
Highest quartile	1073 (24.8)	5416 (25)
Missing	30 (0.7)	326 (1.5)
Family income		
Low	1825 (30.1)	7353 (24.3)
Medium-low	1668 (27.5)	7521 (24.8)
Medium-high	1318 (21.7)	7526 (24.8)
High	1216 (20.1)	7455 (24.6)
Missing	33 (0.5)	445 (1.5)
FIGO stage		
IA	1322 (21.8)	NA
IB	2020 (33.3)	NA
II+	1872 (30.9)	NA
Missing	846 (14.0)	NA
Histopathology type		
Squamous cell cancer	4256 (70.2)	NA
Adenocarcinoma	1385 (22.9)	NA
Other types	419 (6.9)	NA

Characteristics of cervical cancer cases diagnosed between 2006 and 2018, and population comparators matched on age, birth year, and region. All socioeconomic variables are measured 1 year before the index/matching date.

a Denmark, Norway, Finland, and Iceland.

b Registered partner indicates same-sex marriage partner.

### Association between cervical cancer and mental disorders in women

**Figure 1.**
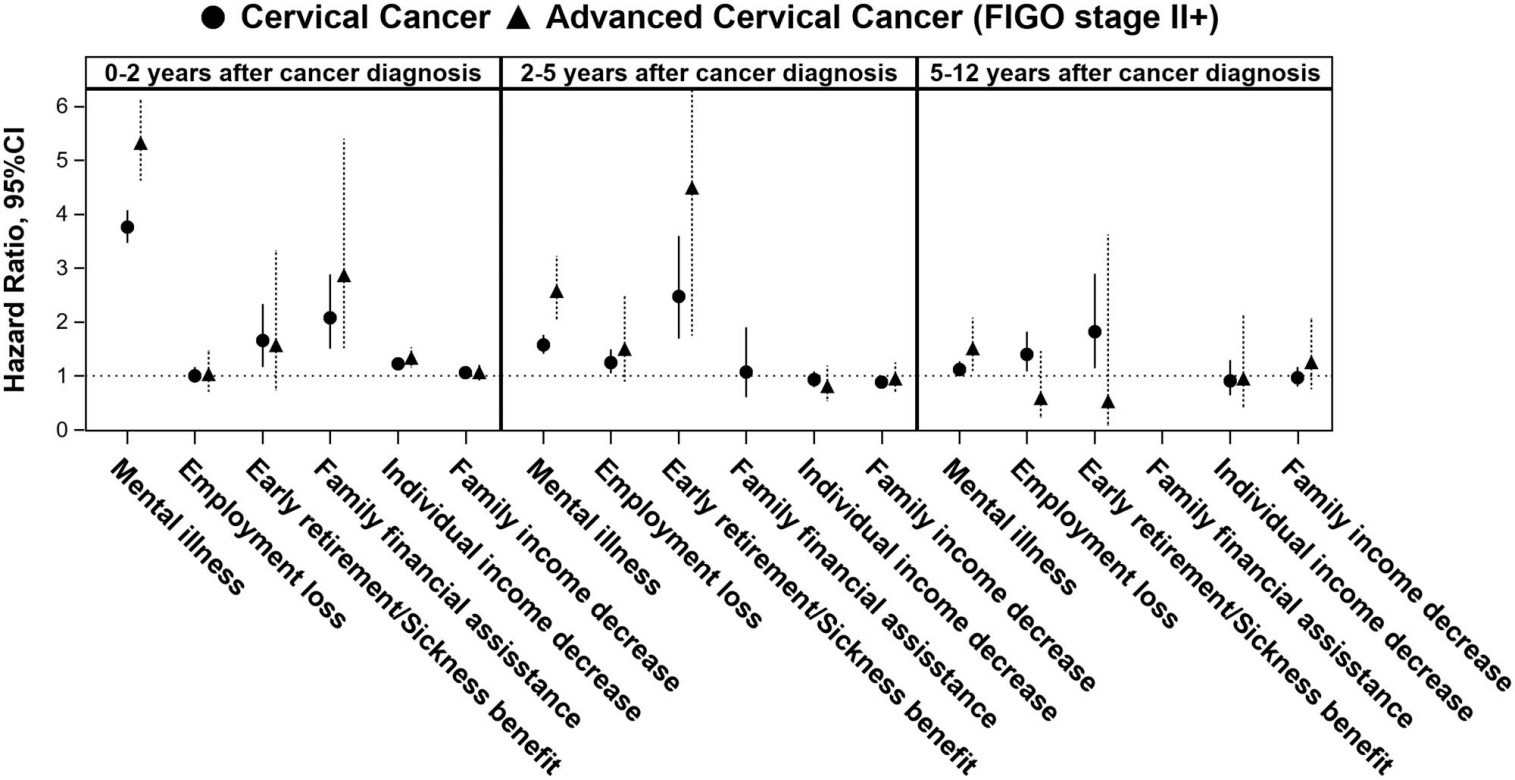
Individual sub-study. Risk for incident mental disorders and socioeconomic changes in cervical cancer cases vs matched comparators (*N* cases = 6060, *N* comparators = 30 300), by time period after index date and severity of diagnosis (all cases, and advanced cases only, respectively). Hazard ratios were estimated with 95% confidence intervals (CIs) adjusted for country of birth and level of education 1 year before index date (see also Tables S1 and S2).

We observed an increased risk of mental disorders associated with cervical cancer diagnosis, particularly in the first 0-2 years after diagnosis (aHR = 3.74, 95% CI = 3.45 to 4.06) (Figure 1, Table S4). The increased risk for mental disorders was more pronounced in women diagnosed with FIGO stage II+, ie, advanced cervical cancer (aHR = 5.32, 95% CI = 4.63 to 6.12 during 0-2 years after cancer diagnosis, Figure 1, Table S5). Particularly high risk was found for tobacco abuse, depression, anxiety, and stress-related disorders (Table S6).

### Association between cervical cancer and socioeconomic outcomes in women

There was an increased risk for unemployment, need for early retirement/sickness benefit, need for family financial support, and decrease in individual income among women with cervical cancer compared with their matched comparators. An increase in the need for early retirement/sickness benefit, need for family financial support, and decrease in individual income appeared immediately after cancer diagnosis, whereas the risk of loss of employment appeared later, from 2 years after cancer diagnosis and became highest during 5-12 years after cancer diagnosis (Figure 1, Table S4).

### Association between cervical cancer and mental disorders in co-parents and children

Among the co-parents of the cases, we observed an increased risk for mental disorders within 2-5 years after index date, compared with the co-parents of the comparators (aHR = 1.32, 95% CI = 1.11 to 1.57, Table 2). This association was modified by vital status of the proband (case/comparator), such that the increased risk was concentrated to the co-parents where the proband died during follow-up (aHR = 1.83, 95% CI = 1.28 to 2.63; *P* for interaction = .0073). When examining potential age-related variation in the increased risk for mental disorders, we observed no statistically significant modification by the age of the co-parent at the index (diagnosis) date (Table 2).

**Table 2.** Family sub-study.

	0-<2 years after cancer diagnosis				2-<5 years after cancer diagnosis				5-<12 years after cancer diagnosis			
Outcomes	*N*	Incidence rate per 1000 person-years	HR (95% CI)	Adjusted HR (95% CI)^a^	*N*	Incidence rate per 1000 person-years	HR (95% CI)	Adjusted HR (95% CI)^a^	*N*	Incidence rate per 1000 person-years	HR (95% CI)	Adjusted HR (95% CI)^a^
**Any mental disorder**												
Overall												
Co-parents of cases	189	30.7	1.20 (1.00 to 1.44)	1.19 (0.98 to 1.44)	225	28.6	**1.24 (1.05 to 1.47)**	**1.32 (1.11 to 1.57)**	230	21.8	0.99 (0.84 to 1.16)	1.03 (0.87 to 1.23)
Co-parents of comparators	768	29.2	Referent	Referent	900	24.8	Referent	Referent	1179	23.6	Referent	Referent
By vital status of the proband woman^b^												
Deceased												
Co-parents of cases	26	32.6	1.38 (0.86 to 2.21)	1.59 (0.98 to 2.58)	55	31.8	**1.52 (1.08 to 2.14)**	**1.83 (1.28 to 2.63)**	62	19.4	0.93 (0.68 to 1.27)	1.13 (0.81 to 1.59)
Co-parents of comparators	11	27.2	Referent	Referent	37	25.5	Referent	Referent	67	14.8	Referent	Referent
Alive												
Co-parents of cases	163	30.4	1.05 (0.86 to 1.27)	1.05 (0.86 to 1.28)	170	27.7	1.11 (0.92 to 1.34)	1.16 (0.96 to 1.41)	129	22.9	0.91 (0.73 to 1.12)	0.92 (0.74 to 1.15)
Co-parents of comparators	757	29.2	Referent	Referent	863	24.8	Referent	Referent	869	25.0	Referent	Referent
By age at index date of the proband woman^c^												
<40												
Co-parents of cases	36	29.5	0.94 (0.62 to 1.43)	0.89 (0.58 to 1.36)	54	31.8	1.28 (0.90 to 1.82)	1.22 (0.85 to 1.74)	47	20.3	0.96 (0.66 to 1.40)	0.96 (0.66 to 1.40)
Co-parents of comparators	151	27.4	Referent	Referent	166	21.6	Referent	Referent	250	22.6	Referent	Referent
41-60												
Co-parents of cases	83	31.9	1.30 (0.99 to 1.71)	1.20 (0.90 to 1.58)	102	29.4	**1.35 (1.06 to 1.73)**	1.27 (0.98 to 1.64)	109	23.8	1.04 (0.83 to 1.31)	0.98 (0.78 to 1.25)
Co-parents of comparators	316	26.4	Referent	Referent	359	21.7	Referent	Referent	474	21.1	Referent	Referent
61-80												
Co-parents of cases	55	34.4	0.89 (0.63 to 1.25)	0.84 (0.58 to 1.20)	59	31.3	1.06 (0.77 to 1.47)	1.03 (0.72 to 1.46)	68	28.8	1.04 (0.76 to 1.42)	1 (0.71 to 1.41)
Co-parents of comparators	225	34.3	Referent	Referent	294	33.3	Referent	Referent	407	35.8	Referent	Referent
81+												
Co-parents of cases	15	20.4	1.04 (0.52 to 2.11)	2.05 (0.83 to 5.09)	10	12.3	0.51 (0.24 to 1.10)	1.03 (0.42 to 2.51)	6	4.7	0.17 (0.05 to 0.59)	0.43 (0.06 to 3.00)
Co-parents of comparators	76	33.1	Referent	Referent	81	25.2	Referent	Referent	48	9.3	Referent	Referent

Risk for incident mental disorders in co-parents to cervical cancer cases vs co-parents to comparators (*N* co-parents of cases = 9272, *N* co-parents of comparators = 43 406), by time period after cancer diagnosis (index) date. Hazard ratios (HRs) were estimated with 95% confidence intervals (CIs), crude and adjusted for individual age, gender, education level and country of birth, family income level, and proband’s educational level and age at diagnosis (index) date. Statistically significant associations are marked in bold.

a Adjusted for co-parents’ level of education, family income, and co-parents’ age at the cancer diagnosis of proband women.

b *P* for interaction = .0073.

c Adjusted further for vital status of the proband cases as a time-varying covariate.

**Figure 2.**
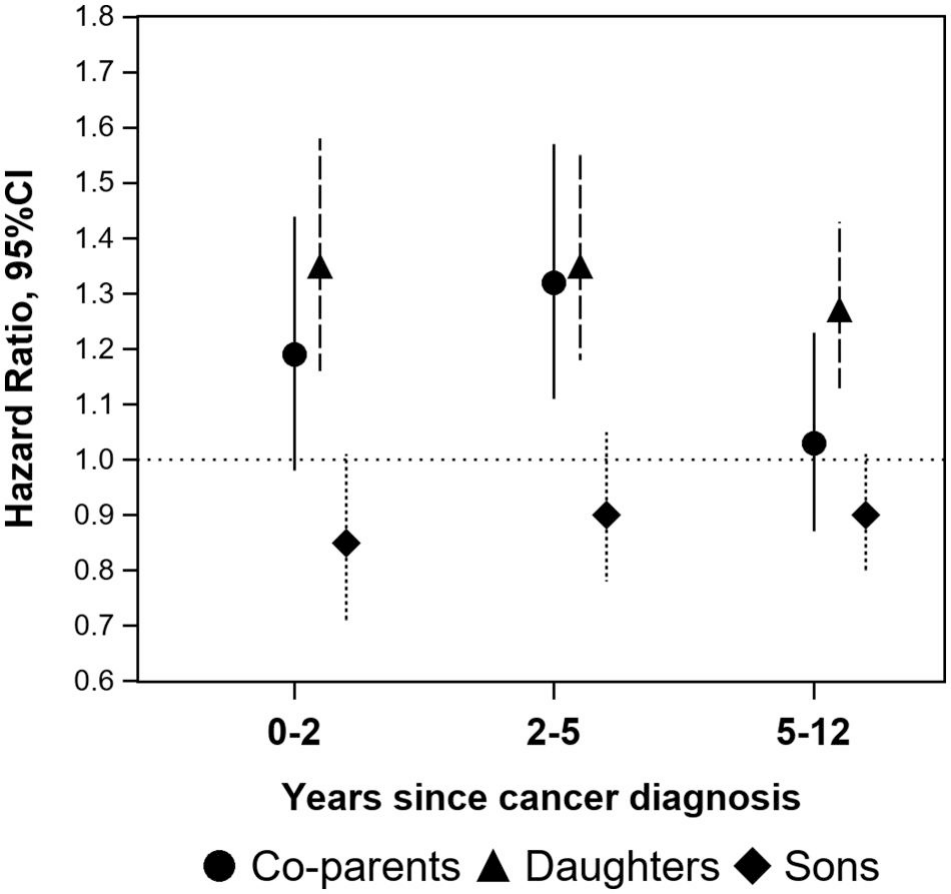
Family sub-study. Risk for incident mental disorder in co-parents or children of cervical cancer cases vs that in co-parents or children of comparators (*N* co-parents of cases = 9272, *N* co-parents of comparators = 43 406, *N* children of cases = 20 435, N children of comparators = 98 600) by time period after cancer diagnosis (index) date and sex of child (where applicable). Hazard ratios were estimated with 95% confidence intervals (CIs). For co-parents, hazard ratios were adjusted for individual age, gender, education level and country of birth, family income level, and proband’s educational level and age at diagnosis (index) date. For children, hazard ratios were adjusted for individual year of birth and age at index date, family income level, and mother’s educational level and age at diagnosis (index) date.

Among the children of the cases, we observed an increased risk for mental disorders immediately after cancer diagnosis, compared with the children of comparators (aHRs = 1.19-1.22 from 0 to 5 years after cancer diagnosis; Figure 2 and Table 3). There was no statistically significant interaction between cervical cancer diagnosis and the vital status of the mother (*P* for interaction > .05). Children at a lower age at their mother’s cancer diagnosis displayed a greater risk increase, compared with children at a higher age (Table 3). Furthermore, we observed an effect modification by gender of the child such that daughters displayed a statistically significant increased risk whereas sons did not (*P* for interaction < .0001). The increased risk of mental disorders among daughters of cases persisted during the entire follow-up period (aHRs = 1.27-1.35 from 0 to 10+ years after diagnosis) (Table 3).

**Table 3.** Family sub-study.

	0-<2 years after cancer diagnosis				2-<5 years after cancer diagnosis				5-<12 years after cancer diagnosis			
Outcomes	*N*	Incidence rate per 1000 person-years	HR (95% CI)	Adjusted HR (95% CI)^a^	*N*	Incidence rate per 1000 person-years	HR (95% CI)	Adjusted HR (95% CI)^a^	*N*	Incidence rate per 1000 person-years	HR (95% CI)	Adjusted HR (95% CI)^a^
**Any mental disorders**												
Overall												
Child of case	387	24.6	**1.26 (1.12 to 1.42)**	**1.22 (1.08 to 1.37)**	533	26.7	**1.21 (1.09 to 1.34)**	**1.19 (1.08 to 1.32)**	767	28.6	1.1 (1.01 to 1.19)	1.08 (0.99 to 1.18)
Child of comparator	1528	22.2	Referent	Referent	2233	23.1	Referent	Referent	3504	25.6	Referent	Referent
By death of the mother												
Yes												
Child of case	49	20.2	0.94 (0.69 to 1.29)	0.91 (0.66 to 1.24)	129	25.3	1.07 (0.87 to 1.30)	1.05 (0.86 to 1.28)	251	27	1.15 (1 to 1.33)	1.14 (0.98 to 1.32)
Child of comparator	30	20.5	Referent	Referent	110	23.1	Referent	Referent	314	20.3	Referent	Referent
No												
Child of case	338	25.5	1.13 (1.00 to 1.28)	1.09 (0.96 to 1.23)	404	27.2	**1.17 (1.04 to 1.31)**	**1.14 (1.01 to 1.28)**	516	29.5	1.07 (0.97 to 1.19)	1.05 (0.95 to 1.17)
Child of comparator	1498	22.2	Referent	Referent	2123	23.1	Referent	Referent	3190	26.2	Referent	Referent
By age of the child at the index date of mother^b^												
<=18												
Child of case	84	13.6	1.01 (0.80 to 1.29)	1.10 (0.86 to 1.41)	199	23.1	**1.26 (1.07 to 1.49)**	**1.35 (1.15 to 1.60)**	336	29.8	1.09 (0.96 to 1.23)	1.1 (0.97 to 1.25)
Child of comparator	375	12.2	Referent	Referent	752	17.2	Referent	Referent	1584	26.7	Referent	Referent
19-30												
Child of case	121	44.0	**1.72 (1.39 to 2.13)**	**1.49 (1.20 to 1.85)**	143	41.1	**1.42 (1.17 to 1.71)**	**1.23 (1.01 to 1.49)**	150	34	1.09 (0.91 to 1.31)	1 (0.83 to 1.20)
Child of comparator	408	30.4	Referent	Referent	591	32.7	Referent	Referent	790	33.2	Referent	Referent
31-50												
Child of case	128	30.7	1.02 (0.83 to 1.24)	1.02 (0.83 to 1.25)	127	25.1	0.88 (0.72 to 1.07)	0.84 (0.68 to 1.03)	180	24.4	1.01 (0.85 to 1.2)	0.98 (0.83 to 1.17)
Child of comparator	499	30.0	Referent	Referent	618	26.7	Referent	Referent	790	21.6	Referent	Referent
51+												
Child of case	54	20.5	**0.73 (0.53 to 0.99)**	**0.71 (0.52 to 0.97)**	64	23.0	0.99 (0.74 to 1.31)	0.93 (0.7 to 1.25)	101	26.9	**1.36 (1.07 to 1.72)**	**1.29 (1.02 to 1.64)**
Child of comparator	246	29.8	Referent	Referent	272	23.3	Referent	Referent	340	19.7	Referent	Referent
By sex of child^c^												
Daughters												
Child of case	222	30.4	**1.38 (1.19 to 1.61)**	**1.35 (1.16 to 1.58)**	301	32.9	**1.39 (1.22 to 1.58)**	**1.35 (1.18 to 1.55)**	409	35	**1.31 (1.17 to 1.47)**	**1.27 (1.13 to 1.43)**
Child of comparator	858	26.5	Referent	Referent	1248	27.9	Referent	Referent	1942	31.5	Referent	Referent
Sons												
Child of case	165	19.7	0.87 (0.73 to 1.03)	0.85 (0.71 to 1.01)	232	21.5	0.93 (0.81 to 1.08)	0.90 (0.78 to 1.05)	358	23.7	0.92 (0.82 to 1.04)	0.9 (0.80 to 1.01)
Child of comparator	670	18.3	Referent	Referent	985	19.0	Referent	Referent	1562	20.8	Referent	Referent

Risk for incident mental disorders in children of cervical cancer cases vs children of comparators (N children of cases = 20 435, N children of comparators = 98 600), by time period after mother’s cancer diagnosis (index) date. Hazard ratios (HR) estimated with 95% confidence intervals (CI), crude and adjusted for individual year of birth and age at index date, family income level, and mother’s educational level and age at diagnosis (index) date. Statistically significant associations are marked in bold.

a Adjusted for family income, and individual (child’s) level of education and age at the index date.

b Adjusted further for vital status of the proband cases as a time-varying covariate.

c Adjusted further for vital status of the proband cases as a time-varying covariate. *P* for interaction < .0001.

### Association between cervical cancer and socioeconomic outcomes in co-parents and children

Co-parents of cases exhibited a slightly increased risk for loss of employment from 0 to 5 years following the cancer diagnosis (aHR = 1.17, 95% CI 1.02-1.34), as well as increased risks for early retirement, need of family financial assistance, and decrease of family income in the first 2 years following the cancer diagnosis (Table 4).

**Table 4.** Family sub-study.

	0-<2 years after cancer diagnosis				2-<5 years after cancer diagnosis				5+ years after cancer diagnosis			
Outcomes	N	Incidence rate per 1000 person-years	HR (95% CI)	Adjusted HR (95% CI)^a^	N	Incidence rate per 1000 person-years	HR (95% CI)	Adjusted HR (95% CI)^a^	N	Incidence rate per 1000 person-years	HR (95% CI)	Adjusted HR (95% CI)^a^
Loss of employment^b^												
Co-parents of cases	359	29.1	**1.15 (1.01 to 1.30)**	**1.17 (1.03 to 1.33)**	307	18.3	**1.19 (1.04 to 1.37)**	**1.17 (1.02 to 1.34)**	479	10	1.06 (0.95 to 1.19)	1.04 (0.93 to 1.16)
Co-parents of comparators	1512	24.6	Referent	Referent	1263	14.9	Referent	Referent	2225	9	Referent	Referent
Early retirement/sickness benefit^c^												
Co-parents of cases	106	7.4	**1.36 (1.08 to 1.72)**	**1.34 (1.06 to 1.71)**	116	5.7	1.12 (0.90 to 1.38)	1.12 (0.89 to 1.40)	308	4.5	**1.26 (1.10 to 1.44)**	**1.26 (1.09 to 1.45)**
Co-parents of comparators	401	5.6	Referent	Referent	514	5	Referent	Referent	1173	3.4	Referent	Referent
Family financial assistance (2011+)^d^												
Co-parents of cases	38	12.6	**1.73 (1.13 to 2.64)**	**1.64 (1.03 to 2.61)**	22	5.2	**1.26 (0.76 to 2.09)**	**1.31 (0.77 to 2.23)**	13	3.9	1.17 (0.60 to 2.30)	1.30 (0.61 to 2.77)
Co-parents of comparators	81	5.5	Referent	Referent	85	4	Referent	Referent	47	2.8	Referent	Referent
Individual income decrease (2009+)												
Co-parents of cases	467	123.5	0.99 (0.89 to 1.11)	0.97 (0.87 to 1.09)	232	51.2	0.99 (0.84 to 1.16)	0.97 (0.83 to 1.15)	124	30.4	1.06 (0.85 to 1.34)	1.04 (0.82 to 1.31)
Co-parents of comparators	2177	118.7	Referent	Referent	1158	52.8	Referent	Referent	562	28.3	Referent	Referent
Family income decrease												
Co-parents of cases	1855	138.3	**1.13 (1.07 to 1.20)**	**1.12 (1.06 to 1.19)**	1020	66.6	1.05 (0.98 to 1.14)	1.05 (0.97 to 1.14)	1146	30	0.95 (0.88 to 1.03)	0.96 (0.89 to 1.03)
Co-parents of comparators	7922	119.1	Referent	Referent	4969	63.6	Referent	Referent	6250	31.9	Referent	Referent

Risk for (negative) socioeconomic outcomes in co-parents to cervical cancer cases vs co-parents to comparators (*N* co-parents of cases = 9272, *N* co-parents of comparators = 43 406), by time period after cancer diagnosis (index) date. Hazard ratios (HRs) estimated with 95% confidence intervals (CIs), crude and adjusted for individual age, gender, education level and country of birth, family income level, and proband’s educational level and age at diagnosis (index) date. Statistically significant associations are marked in bold.

a Adjusted for individual country of birth, educational level, and age at index date.

b Among co-parents employed 1 year before index date.

c Among co-parents with neither early retirement nor sickness benefit 1 year before index date.

d Among co-parents without family financial assistance 1 year before index date.

Among children who were aged 0-18 at cancer diagnosis of their mother, and who were 19 years or older at the end of follow-up, we observed a lower likelihood of attaining a higher educational level in adult life, compared with children of comparators (odds ratio = 0.87, 95% CI 0.83-0.91, Table 5).

**Table 5.** Family sub-study.

Highest educational attainment defined by Statistics Sweden terminology (No. of years)	Children of cases (%)	Children of comparators (%)	Odds ratio for higher education level in children of cases vs children of comparators (95% CI)^a^
Primary school education for less than 9 years	25 (0.43)	66 (0.22)	**0.87 (0.83 to 0.91)**
Primary school education for full 9 years	1228 (21.23)	5465 (18.49)	
Upper secondary school/high school studies for 2 years	317 (5.48)	1233 (4.17)	
Upper secondary school/high school studies for 3 years	2419 (41.82)	11 923 (40.35)	
College/university/special professional training for less than 3 years	768 (13.28)	4398 (14.88)	
College/university/special professional training for 3 years or longer (excl. postgraduate education)	1015 (17.55)	6370 (21.56)	
Postgraduate education	13 (0.22)	97 (0.33)	
**Total**	**5785**	**29 552**	

Association between higher educational attainment and being the child of a cervical cancer case vs the child of a comparator. Odds ratio estimated with 95% confidence interval (CI), for children who were younger than age 19 at the index date. Statistically significant associations are marked in bold.

a Estimated by conditional ordinal logistic regression model, adjusted for mother’s education and family income at index date, child’s age at the end of follow-up, and child’s year of birth.

## Discussion

We recently found that women with prevalent mental disorders participate less in cervical screening and experience a higher risk of cervical cancer.^11^ We here focus on risk of incident mental disorders *after* actual diagnosis of cervical cancer and find that this is elevated also in women with no previous psychiatric history. This may be an expected finding, given the negative impact of receipt of such a diagnosis. What is less known, however, is the potential duration of this risk increase, and corresponding risks among the woman’s immediate family members; her co-parent(s) and children. We here describe potential negative mental and socioeconomic impact that persist over time and across generations; highlighting problematic trends in both mental health and education level outcomes in children, perhaps particularly in daughters.

There are several studies looking into the mental well-being of cervical cancer patients, but few are based on national cohorts. Horsboel et al. followed women diagnosed with ovarian, endometrial, or cervical cancer in Denmark during 1998-2013 and investigated use of antidepressants compared with matched references.^12^ In line with our findings, the cumulative risk of antidepressant use during a period of 10 years after diagnosis showed a higher increase in the first few years after diagnosis of cervical cancer, and then equalized with the reference group. The risk for use of antidepressants during the first year after diagnosis was 3-fold increased in the cohort of 3758 cervical cancer patients (95% CI = 2.74 to 3.61). Kim et al. followed 36 801 women diagnosed with breast, cervical, endometrial, or ovarian cancer in Korea between 2010 and 2020 to investigate incidence and risk of developing mental health problems. However, the study lacked reference to matched comparators. During a median follow-up of 5.6 years, almost half of the 4873 included patients with cervical cancer developed an anxiety disorder after their diagnosis, whereas a fifth developed a depressive disorder.^13^ Our results mirror these, in that anxiety was more common postdiagnosis than overt depression.

Comprehensive studies on co-parents/spouses and children of cervical cancer patients are scarce and to our knowledge this is the first study to follow a national cohort of children of cervical cancer patients. We previously investigated if spouses of patients with cancer (of all types) had an increased risk of developing mental disorders and using national registries in Denmark and Sweden.^11^ During the 8-year long follow-up we found that spouses of patients with cervical cancer had a 23% increased risk of developing a new mental disorder compared with others. The present study showed a risk increase in any mental disorders in co-parents of patients with cervical cancer during 2-5 years after cancer diagnosis, which is in line with the previous findings in spouses. Several studies have used questionnaires to collect data from caregivers (male partners, daughters, etc) reporting an increase in psychological distress and economic burden on the families of cervical cancer patients responsible for informal care in lower resource settings.^7-9^ However, our study is the first national cohort to report in depth on socioeconomic consequences among co-parents/spouses in a high-resource setting and the first to report on negative outcomes in mental well-being and educational attainment of children to women with cervical cancer. A cancer diagnosis in a parent, which includes a threat of death, induces stress in children, and may lead to posttraumatic stress disorder and long-term psychosocial impact.^14-16^

We found similar lower educational attainment among all children, but more pronounced mental health problems in daughters rather than sons, implying a potential gender difference in the latter. The literature suggests that there may be true gender differences in distress level vs coping skills, but that these are challenging to disentangle (reviewed in Zhou et al^17^), and in this quantitative study, we cannot delve deeply into explanations for this finding. However, the higher risk of mental health problems in daughters carries the caveat that such results may in part be due to (mental) healthcare seeking behavior being higher among female compared with male children over the duration of this register-based study. Thus, some caution is warranted when interpreting this finding, as there could be some underdiagnosis in male children if these are not properly assessed. Accordingly, we posit that negative development in all children is a potential risk and that boys and girls both naturally should be equally and actively monitored. Indeed, psychosocial interventions used today may not address anxiety and behavioral problems in children adequately^18^ and it may be suitable to increase routines for surveillance and long-term active follow-up of these children’s health, particularly if the mother passes away from the disease.

Regarding the co-parents, the financial toxicity that cancer can bring to a household has been illuminated recently, in studies showing how the negative mental impact of a role as a caregiver has consequences on both health and ability to work.^19^ This could be highly relevant for outcomes in co-parents in this study. However, we did not detect any long-term substantial declines in household income, indicating that the social support system in Sweden may be substantial enough to counteract detrimental effects on family income through, for example, financial assistance. We posit that negative financial outcomes in co-parents and children may be even more pronounced in settings where such support is not available.

The strengths of our study include the complete inclusion of cases and high-quality definitions of exposures and outcomes inherent to the national registry infrastructure of Sweden. The Multi-Generation Register together with the Swedish Cancer Register offered unbiased familial information not accessible through self-report, particularly among women who died. This yielded comprehensive and minimally biased data. The limitations of our study include the observational design which precludes causal inference, as the noted associations may be due not only to cervical cancer status but also to genetic factors (related to risk of both cervical cancer and mental disorders) and/or lifestyle choices (related to risk of both cervical cancer and differential educational attainment). However, the observed temporal pattern of the results argues against such as the sole explanation of our findings. Also, the number of events in some sub-strata were small, which limited the strength of conclusions in sub-group analyses.

Another limitation was that we had suboptimal possibility to study the potential effect modification of civil status on outcomes after cancer. Our data do contain information of marriage/lack of such, divorce, registered partnership and widowhood for all the adults included. However, many Swedish couples may co-habit without being married, because of our secular society and generational trends. Thus, the “unmarried” group would risk being heavily biased by proband women having financial and emotional support from an in-living co-parent. This misclassification would render the analysis less informative and a comprehensive study on co-habitation should be the focus of future work. We also could not study marriage or co-habiting partners to the case and comparator women if they did not share children, due to our main research focus being the definition of co-parents through a common child. Thus, there remains a complementary gap of knowledge regarding mental health and socioeconomic outcomes in child-less couples after a cervical cancer diagnosis. Further research is needed to characterize outcomes in such couples, particularly as cervical cancer can affect fertility through invasive treatment. Finally, our approach focused on downward changes in socioeconomic categories after index date compared with that in the year before. Ergo, those few study participants who were simultaneously in the lowest referral level of all categories could not be analyzed for a further negative trend. These most deprived participants should therefore be the focus of further complementary work.

Our findings emphasize the need for focused psychosocial support for women and family members that persist over time—not just immediately after the woman’s diagnosis, and particularly if the child is a minor at the mother’s diagnosis, and/or if the cancer is advanced-stage or fatal—findings that extend some previous reports.^11^^,^^20^ Although the observational nature of this study precludes formal causal inference, we would suggest that a long-term structured plan for how to actively work with family health and mitigate negative outcomes could be a standard part of the patient care pathway. Such efforts could be included in quality-of-care evaluations and may be helpful to reduce potential negative cross-generational impact.

## Conclusions

Incident mental disorders and socioeconomic adverse outcomes are more common after diagnosis among women with cervical cancer, the adults with whom they co-parent, and their children, than previously known. Adults who co-parent with women with cervical cancer display negative trends in outcomes postdiagnosis of their partner. These negative trends were most apparent in family members where the woman died. Daughters to mothers with cervical cancer are at increased risk of mental disorders and both daughters and sons are at risk for lower educational attainment. Young age of the child at mother’s diagnosis is an additional risk factor.

## Supplementary Material

djaf129_Supplementary_Data

## Data Availability

The data underlying the publication is subject to access restrictions according to Swedish data privacy law and the EU GDPR, and thus, the authors are not able to make the individual-level dataset publicly available as it contains pseudonymized sensitive health data. The source data used in this study are retrievable from Statistics Sweden (SCB) and the Swedish National Board of Health and Welfare (Socialstyrelsen). Any researchers, including international researchers, who are interested in obtaining the data can contact SCB via information@scb.se, and Socialstyrelsen via registerservice@socialstyrelsen.se. One can contact: https://www.scb.se/om-scb/kontakta-oss/statistikservice/fraga-oss (SCB) and visit https://bestalladata.socialstyrelsen.se/data-for-forskning/ (Socialstyrelsen) for detailed information about how to apply for access to register data for research purposes. An ethical permission is required, obtained either through the researcher’s own institution, or through an application in Swedish to the Ethical Review Authority of Sweden.
