# Supplementary material for: Mental disorders and socioeconomic outcomes in women with cervical cancer, and their children and co-parents
Source: J Natl Cancer Inst. 2025 Jun 10;117(9):1825–35. doi: 10.1093/jnci/djaf129 (PMC12415958; doi:10.1093/jnci/djaf129)
Supplement: djaf129_Supplementary_Data [file djaf129_supplementary_data.pdf]

SUPPLEMENTARY MATERIAL

SUPPLEMENTARY METHODS

Data sources

Swedish Cancer Register

The Swedish Cancer Register was established in 1958 and obtains mandatory reported data from both clinicians and pathologists on all newly diagnosed malignant neoplasms. The register provides the basis for official cancer statistics in Sweden (National Board of Health and Welfare, SCR, 2024). The register includes data on patients (e.g., gender, age), medical data (e.g., site of tumor, histology) and follow-up data (e.g., date of death, cause of death). The register is considered to be of high quality as approximately 99% of the cases are morphologically verified; a study from 2008 reported an underreporting of approximately 4% (Barlow et al, 2009).

National Patient Register (NPR)

Diseases and surgical procedures have been registered in NPR since the 1960's. From 1987 the NPR includes all in-patient and from 2001 also majority of out-patient care delivered in Sweden. The register includes patient data (e.g., gender, age), geographical data (e.g., county council, hospital), administrative data (e.g., inpatient and outpatient data) and medical data (e.g., discharge diagnosis) (Ludvigsson et al, 2011; National Board of Health and Welfare, NPR, 2024). Primary care is not covered in the NPR.

Swedish Prescribed Drug Register (PDR)

The Swedish Prescribed Drug Register was established in 2005 containing all prescribed drugs dispensed at the pharmacies in the nation, whether originating from specialist or primary care prescribers (Wettermark et al, 2007). Medicines that are administrated in hospitals or at nursing homes as well as over-the-counter medications are not included in the register.

The register includes data concerning the patient (e.g., gender, age), product (e.g., ATC code, drug name, strength), costs, and the prescribing clinician (National Board of Health and Welfare, PDR, 2024).

Causes of Death Register (COD)

The Causes of Death Register contains all deaths in the Swedish population (National Board of Health and Welfare, COD, 2024). A physician needs to sign a death certificate including information on causes of death for each death (Brooke et al, 2017).

Multi-Generation Register

The Multi-Generation Register is a part of the Total Population Register which is based on the National Tax Board at Statistics Sweden. The register consists of all persons who have been registered as a resident in Sweden since 1961, who were born in 1932 or later, and the connection between persons and their biological or adoptive parents (Ekbom, 2011).

Longitudinal Integrated database for Health insurance and Labour market studies (LISA)

Statistics Sweden's longitudinal database, LISA, includes the Swedish population aged ≥16 years and since 2010 individuals aged ≥15 years. Registration in LISA is compulsory and includes data on demography, education, employment and unemployment, income and social insurance, civil status, workplace and company. Data on occupation have a completeness of 95% and >98% of education data are available for individuals aged 25-64 years (Ludvigsson et al, 2019).

Definition of socioeconomic changes

Loss of employment

Employment status was recorded in Longitudinal Integrated database for Health insurance and Labour market studies (LISA) every year. It records whether there is any unemployment that happened within a specific year. Loss of employment in this study is defined as: among women with no unemployment in the year before cancer diagnosis (or selection as comparators), the first year the women started to have a record of unemployment.

Early retirement/sickness benefit

LISA has record of whether an individual has an early retirement, or receives sickness allowance, sickness compensation, or activity compensation each year. In this study, this indicator is defined as: among women with no early retirement or not receiving the mentioned allowance/compensation in the year before cancer diagnosis (or selection as comparators), the first year the women started to receive them.

Family finance assistance

LISA records this since year 2011. It identifies individuals belonging to a family that received financial assistance in a specific year. This assistance is given to the ones whose income cannot afford housing expenses. In this study, this indicator is defined as: among women receiving no family finance assistance in the year before cancer diagnosis (or selection as comparators), the first year one receives it.

SUPPLEMENTARY REFERENCES

Barlow L et al. The completeness of the Swedish Cancer Register - a sample survey for year 1998. Acta Oncologica, 2009; 48: 27-33

Brooke HL, Talbäck M, Hörnblad J, Johansson LA, Ludvigsson JF, Druid H, Feychting M, Ljung R. The Swedish cause of death register. Eur J Epidemiol. 2017 Sep;32(9):765-773. doi: 10.1007/s10654-017-0316-1. Epub 2017 Oct 5. PMID: 28983736; PMCID: PMC5662659.

Ekbom A. The Swedish Multi-generation Register. Methods Mol Biol. 2011;675:215-20. doi: 10.1007/978-1-59745-423-0_10. PMID: 20949391.

Ludvigsson, J.F., Svedberg, P., Olén, O. et al. The longitudinal integrated database for health insurance and labour market studies (LISA) and its use in medical research. Eur J Epidemiol 34, 423-437 (2019). https://doi.org/10.1007/s10654-019-00511-8

National Board of Health and Welfare. National Cancer Register. Available at https://www.socialstyrelsen.se/en/statistics-and-data/registers/national-cancer-register/ (last accessed on 25 June 2024)

National Board of Health and Welfare. National Cause of Death Register. Available at https://www.socialstyrelsen.se/en/statistics-and-data/registers/national-cause-of-death--register/ (last accessed on 25 June 2024)

National Board of Health and Welfare. National Patient Register. Available at https://www.socialstyrelsen.se/en/statistics-and-data/registers/national-patient-register/ (last accessed on 25 June 2024)

National Board of Health and Welfare. National Prescribed Drug Register. Available at https://www.socialstyrelsen.se/en/statistics-and-data/registers/national-prescribed-drug-register/ (last accessed on 25 June 2024)

Wettermark B, Hammar N, Fored CM, Leimanis A, Otterblad Olausson P, Bergman U, Persson I, Sundström A, Westerholm B, Rosén M. The new Swedish Prescribed Drug Register--opportunities for pharmacoepidemiological research and experience from the first six months. Pharmacoepidemiol

Drug Saf. 2007 Jul;16(7):726-35. doi: 10.1002/pds.1294. Erratum in: Pharmacoepidemiol Drug Saf. 2008 May;17(5):533. MichaelFored, C [corrected to Fored, Carl Michael]. PMID: 16897791.

SUPPLEMENTARY TABLES

Table S1. Included mental disorders and corresponding ICD-10 code

**Category**	**ICD-8 (1968-1986)**	**ICD-9 (1987-1996)**	**ICD-10 (1997-2018)**
Any mental disorder	291, 295-315	291, 292, 295-319	F10-F99
Substance abuse, excl. tobacco- and alcohol-related disorders	304	292, 303-305 except (305A, 305B)	F10-F19 except (F10, F17)
Alcohol-related disorder	291, 303	305B, 291	F10
Tobacco-related disorder	-	305A	F17
Psychotic disorder	295, 297, 298, 299	295, 297, 298	F20-F29
Depression	296.0, 296.2, 298.0, 300.4	296B, 296D, 298A, 300E, 311	F32-F33
Anxiety	300.0, 300.2	300A, 300C	F40-F41
Stress-related disorder	307	308, 309	F43

Table S2: Characteristics of co-parents to proband women (cases or comparators, respectively)

	**Cases (N)**	**(% column)**	**Comparators (N)**	**(% column)**	**P value by chi-square**
Number of co-parents for each proband					
0	2381	20,5	11523	19,9	<0,0001
1	7691	66,3	41752	72,0	
2	1341	11,6	4319	7,4	
3+	189	1,6	416	0,7	
Country of birth of co-parent					
Sweden	9580	88,0	44810	87,1	0,0003
Nordic	394	3,6	1661	3,2	
Other	904	8,3	4886	9,5	
Missing	13	,1	73	,1	
Age of co-parent at index date					
<30	257	2,4	1463	2,8	0,1449
30-39	1618	14,9	7862	15,3	
40-49	2528	23,2	11724	22,8	
50-59	1917	17,6	9077	17,6	
60-69	1539	14,1	7197	14,0	
70-79	1484	13,6	6911	13,4	
80-89	1115	10,2	5260	10,2	
90+	433	4,0	1936	3,8	
Education level of co-parents					
Low (less than 9 years)	2526	23,2	11949	23,2	<0,0001
Middle (high school)	4082	37,5	18856	36,7	
High (college+)	1741	16,0	10557	20,5	
Missing	2542	23,3	10068	19,6	
Family income of co-parent before index date (in quartile)					
Quartile 1	1904	17,5	6591	12,8	<0,0001
Quartile 2	2136	19,6	9437	18,3	
Quartile 3	2210	20,3	12796	24,9	
Quartile 4	2208	20,3	13026	25,3	
Missing	2433	22,3	9580	18,6	

Table S3: Characteristics of <u>children</u> to proband mothers (cases or comparators, respectively)

	**Cases (N)**	**(% column)**	**Comparators**	**(% column)**	**P value by chi-square**
Number of children per proband					
0	2083	18,0	10644	18,3	<0,0001
1	2330	20,1	10936	18,9	
2	4099	35,3	21819	37,6	
3	2012	17,3	10220	17,6	
4+	1078	9,3	4391	7,6	
Origin of children					
Adoptive	211	1,0	1190	1,1	0,0658
Biological	21286	99,0	104573	98,9	
Sex					
Female	10918	50,8	52771	49,9	0,0170
Male	10579	49,2	52992	50,1	
Country of birth of children					
Sweden	20430	95,0	100404	94,9	0,8142
Nordic	169	,8	856	,8	
Other	898	4,2	4503	4,3	
Age of child at mother's index date					
1-5	1526	7,1	8058	7,6	
6-10	2055	9,6	10715	10,1	
11-15	2139	10,0	10896	10,3	
16-20	2038	9,5	10053	9,5	
21-25	1845	8,6	8890	8,4	
26-30	1694	7,9	8087	7,6	
31+	9651	44,9	43662	41,3	
Maximum length of follow-up after mother's index date					
0-4 years	393	1,8	3874	3,7	
5-9 years	114	,5	1221	1,2	
10-14 years	37	,2	272	,3	
15-19 years	5	,0	35	,0	

Table S4: Individual sub-study. Risk of incident mental disorders and (negative) socioeconomic outcomes in cervical cancer cases vs. matched comparators, by time period after cancer diagnosis (index) date (all cases regardless of stage). Hazard ratios were estimated with 95% confidence intervals (CIs). Crude HRs and HRs adjusted for country of birth and level of education one year before index date are presented.

	0-2 years after cancer diagnosis				2-5 years after cancer diagnosis				5-12 years after cancer diagnosis			
Outcomes	N	Incidence rate per 1000 person-years(pys)	HR (95%CI)	Adjusted HR (95% CI)[e]	N	Incidence rate per 1000 pys	HR (95%CI)	Adjusted HR (95% CI)[e]	N	Incidence rate per 1000 pys	HR(95%CI)	Adjusted HR (95% CI)[e]
Any mental disorders[a]												
Cases	1245	215,4	**3.78 (3.48-4.1)**	**3.74 (3.45-4.06)**	503	99,4	**1.59 (1.43-1.78)**	**1.57 (1.41-1.75)**	391	94,9	**1.15 (1.01-1.29)**	1.12 (0.99-1.27)
Comparators	2469	61,8	Ref.	Ref.	2893	70,1	Ref.	Ref.	3232	90,0	Ref.	Ref.
Loss of employment[b]												
Cases	242	28,1	0.97 (0.84-1.11)	1.01 (0.87-1.16)	173	23,1	**1.25 (1.05-1.49)**	**1.25 (1.05-1.5)**	91	14,5	**1.48 (1.16-1.91)**	**1.45 (1.12-1.87)**
Comparators	1248	26,2	Ref.	Ref.	743	15,0	Ref.	Ref.	409	8,9	Ref.	Ref.
Early retirement/sickness benefit[c]												
Cases	50	5,5	**1.80 (1.29-2.51)**	**1.65 (1.17-2.33)**	51	6,0	**2.84 (1.98-4.07)**	**2.51 (1.73-3.65)**	29	3,9	**1.77 (1.14-2.74)**	**1.82 (1.14-2.90)**
Comparators	152	3,0	Ref.	Ref.	107	2,0	Ref.	Ref.	93	1,8	Ref.	Ref.
Family financial assistance (2011+)[d]												
Cases	63	12,5	**1.69 (1.27-2.26)**	**2.09 (1.51-2.88)**	16	4,6	0.95 (0.55-1.64)	1.07 (0.61-1.9)	0	0	-	-
Comparators	198	7,1	Ref.	Ref.	92	4,1	Ref.	Ref.	4	1,1	-	-
Individual income decrease (2009+)												
Cases	924	158,6	**1.27 (1.18-1.36)**	**1.23 (1.14-1.32)**	209	57,2	0.96 (0.82-1.12)	0.93 (0.8-1.09)	46	36,4	0.96 (0.68-1.37)	0.91 (0.64-1.29)
Comparators	3886	118,0	Ref.	Ref.	1442	57,6	Ref.	Ref.	300	33,2	Ref.	Ref.
Family income decrease												
Cases	1067	122,9	1.07 (1.00-1.15)	1.06 (0.99-1.14)	355	52,9	0.91 (0.81-1.02)	0.89 (0.79-1.00)	166	31,2	1.00 (0.84-1.20)	0.98 (0.82-1.18)
Comparators	5378	112,0	Ref.	Ref.	2516	58,2	Ref.	Ref.	1144	31,9	Ref.	Ref.

a. *Any specialist diagnosis of mental disorders (according to the Swedish Patient Register) or any prescribed use of psychiatric medication (according to the Swedish Prescribed Drug Register).*
b. *Among cases and comparators who were employed one year before index date.*
c. *Among cases and comparators with neither early retirement nor sickness benefit one year before index date.*
d. *Among cases and comparators without family financial assistance one year before index date.*
e. *Adjusted for country of birth and educational level.*

Table S5: Individual sub-study. Risk of any incident mental disorder and (negative) socioeconomic outcomes in advanced cervical cancer cases (FIGO stage II+) vs. matched comparators, by time period after cancer diagnosis (index) date. Hazard ratios estimated with 95% confidence intervals (CIs) are presented crude and adjusted for country of birth and level of education one year before index date.

	0-2 years after cancer diagnosis				2-5 years after cancer diagnosis				5-12 years after cancer diagnosis			
Outcomes	N	Incidence rate per 1000 pys	HR (95%CI)	Adjusted HR (95% CI)[e]	N	Incidence rate per 1000 pys	HR (95%CI)	Adjusted HR (95% CI)[e]	N	Incidence rate per 1000 pys	HR(95%CI)	Adjusted HR (95% CI)[e]
Any mental disorder[a]												
Cases	533	395,3	**5.36 (4.67-6.16)**	**5.32 (4.63-6.12)**	146	174,6	**2.58 (2.06-3.23)**	**2.57 (2.05-3.21)**	66	163,1	**1.48 (1.08-2.03)**	**1.52 (1.11-2.09)**
Comparators	917	79,8	Ref.	Ref.	1001	90,3	Ref.	Ref.	924	112,5	Ref.	Ref.
Loss of employment[b]												
Cases	40	17,1	1.08 (0.76-1.52)	1.03 (0.72-1.47)	22	14,8	1.47 (0.90-2.41)	1.50 (0.91-2.48)	7	8,1	0.93 (0.40-2.16)	0.59 (0.24-1.46)
Comparators	214	13,8	Ref.	Ref.	127	8,2	Ref.	Ref.	66	5,3	Ref.	Ref.
Early retirement/sickness benefit[c]												
Cases	11	4,7	1.89 (0.92-3.87)	1.57 (0.74-3.33)	12	7,7	**4.60 (1.97-10.73)**	**4.49 (1.75-11.51)**	2	2,1	0.82 (0.18-3.76)	0.53 (0.08-3.62)
Comparators	35	2,3	Ref.	Ref.	17	1,1	Ref.	Ref.	17	1,4	Ref.	Ref.
Family financial assistance[d]												
Cases	21	14,1	**2.35 (1.39-3.97)**	**2.87 (1.52-5.40)**	0	0	-	-	0	0	-	-
Comparators	52	5,3	Ref.	Ref.	20	2,6			1	0,8		
Individual income decrease												
Cases	261	152,4	**1.34 (1.17-1.54)**	**1.34 (1.16-1.53)**	32	40,3	0.84 (0.57-1.23)	0.80 (0.54-1.19)	9	40,2	1.01 (0.46-2.23)	0.95 (0.42-2.13)
Comparators	1171	103,8	Ref.	Ref.	422	49,8	Ref.	Ref.	92	32,3	Ref.	Ref.
Family income decrease												
Cases	273	119,3	1.06 (0.93-1.21)	1.06 (0.93-1.21)	65	51,7	0.95 (0.72-1.26)	0.94 (0.71-1.25)	25	36,6	1.18 (0.72-1.94)	1.25 (0.75-2.07)
Comparators	1596	107,6	Ref.	Ref.	707	56,3	Ref.	Ref.	293	34,2	Ref.	Ref.

a. *Any specialist diagnosis of mental disorders (according to the Swedish Patient Register) or any prescribed use of psychiatric medication (according to the Swedish Prescribed Drug Register).*
b. *Among cases and comparators who were employed one year before index date.*
c. *Among cases and comparators with neither early retirement nor sickness benefit one year before index date.*
d. *Among cases and comparators without family financial assistance one year before index date.*

e.   Adjusted for country of birth and educational level.

Table S6: Individual sub-study. Risk for specific incident mental disorders among cervical cancer cases vs. comparators, by time period after cancer diagnosis (index) date. Hazard ratios (HR) estimated with 95% confidence intervals (CI), crude and adjusted for country of birth and level of education.

	0-2 years after cancer diagnosis				2-5 years after cancer diagnosis				5-12 years after cancer diagnosis			
**Outcomes**	**N**	**IR per 100,000 py**	**HR (95%CI)**	**Adjusted HR (95% CI)***	**N**	**IR per 100,000 py**	**HR (95%CI)**	**Adjusted HR (95% CI)***	**N**	**IR per 100,000 py**	**HR (95%CI)**	**Adjusted HR (95% CI)***
**Any mental disorder**												
Cases	1245	215,4	**3.78 (3.48-4.1)**	**3.74 (3.45-4.06)**	503	99,4	**1.59 (1.43-1.78)**	**1.57 (1.41-1.75)**	391	94,9	1.15 (1.01-1.29)	1.12 (0.99-1.27)
Comparator	2469	61,8	Ref.	Ref.	2893	70,1	Ref.	Ref.	323 2	90,0	Ref.	Ref.
**Specialist diagnosis of mental disorder**												
Substance abuse												
Cases	11	1,1	1.68 (0.85-3.34)	1.46 (0.72-2.96)	13	1,4	1.60 (0.86-3.01)	1.56 (0.81-2.99)	9	1,1	1.03 (0.50-2.13)	1.03 (0.49-2.15)
Comparators	37	0,7	Ref.	Ref.	49	0,8	Ref.	Ref.	50	0,8	Ref.	Ref.
Alcohol abuse												
Cases	10	1,0	1.06 (0.54-2.11)	0.99 (0.49-1.99)	11	1,2	0.88 (0.46-1.66)	0.89 (0.46-1.7)	15	1,8	1.56 (0.87-2.80)	1.43 (0.79-2.58)
Comparators	55	1,0	Ref.	Ref.	83	1,4	Ref.	Ref.	63	1,1	Ref.	Ref.
Tobacco abuse												
Cases	26	2,7	**5.6 (3.2-9.82)**	**4.81 (2.60-8.89)**	14	1,5	**2.28 (1.21-4.33)**	**2.52 (1.27-4.99)**	5	0,6	0.66 (0.26-1.69)	0.64 (0.24-1.67)
Comparators	29	0,5	Ref.	Ref.	36	0,6	Ref.	Ref.	57	1,0	Ref.	Ref.
Psychotic disorder												
Cases	6	0,6	1.28 (0.52-3.15)	1.32 (0.51-3.41)	9	1,0	1.69 (0.79-3.61)	1.73 (0.79-3.79)	4	0,5	0.83 (0.28-2.40)	0.62 (0.19-1.98)
Comparators	27	0,5	Ref.	Ref.	36	0,6	Ref.	Ref.	32	0,5	Ref.	Ref.
Depression												
Cases	113	12,5	**2.59 (2.05-3.25)**	**2.53 (2.00-3.18)**	70	8,2	**1.63 (1.24-2.15)**	**1.6 (1.22-2.11)**	33	4,2	0.92 (0.63-1.34)	0.93 (0.64-1.36)
Comparators	241	4,6	Ref.	Ref.	282	4,9	Ref.	Ref.	254	4,4	Ref.	Ref.
Anxiety												
Cases	199	21,9	**5.44 (4.44-6.67)**	**5.33 (4.34-6.55)**	72	8,5	**1.73 (1.32-2.26)**	**1.7 (1.30-2.24)**	38	4,8	1.06 (0.75-1.52)	1.10 (0.77-1.57)
Comparators	212	4,0	Ref.	Ref.	281	4,8	Ref.	Ref.	261	4,5	Ref.	Ref.
Stress-related disorders												
Cases	115	12,3	**3.38 (2.66-4.30)**	**3.33 (2.60-4.25)**	50	5,7	**1.69 (1.22-2.33)**	**1.63 (1.18-2.26)**	33	4,1	1.07 (0.74-1.57)	1.04 (0.71-1.52)
Comparators	188	3,5	Ref.	Ref.	189	3,2	Ref.	Ref.	188	3,2	Ref.	Ref.
Other												

Cases	47	5,1	1.33 (0.96-1.83)	1.27 (0.92-1.76)	52	6,0	**1.71 (1.24-2.35)**	**1.64 (1.19-2.27)**	25	3,1	1.00 (0.65-1.55)	1.01 (0.65-1.56)
Comparators	201	3,8	Ref.	Ref.	193	3,3	Ref.	Ref.	167	2,9	Ref.	Ref.
**Prescribed use of psychiatric medications**												
Antidepressants												
Cases	587	66,6	**2.28 (2.07-2.53)**	**2.24 (2.02-2.48)**	415	51,2	**1.53 (1.36-1.71)**	**1.49 (1.33-1.67)**	450	63,1	**1.25 (1.12-1.39)**	**1.21 (1.08-1.35)**
Comparators	1530	30,9	Ref.	Ref.	1857	34,7	Ref.	Ref.	2732	53,8	Ref.	Ref.
Anxiolytics												
Cases	827	95,7	**4.24 (3.85-4.67)**	**4.20 (3.81-4.62)**	384	47,9	**1.99 (1.77-2.25)**	**1.97 (1.74-2.23)**	326	45,7	**1.39 (1.22-1.58)**	**1.36 (1.19-1.54)**
Comparators	1208	24,1	Ref.	Ref.	1432	26,4	Ref.	Ref.	1961	37,8	Ref.	Ref.
Hypnotics/sedatives												
Cases	1003	125,4	**4.48 (4.10-4.90)**	**4.41 (4.03-4.82)**	409	56,1	**1.90 (1.68-2.14)**	**1.89 (1.67-2.12)**	310	48,7	**1.27 (1.11-1.45)**	**1.25 (1.09-1.42)**
Comparators	1460	30,2	Ref.	Ref.	1787	34,7	Ref.	Ref.	2170	45,0	Ref.	Ref.
Antipsychotics												
Cases	152	15,7	**3.65 (2.95-4.52)**	**3.59 (2.89-4.45)**	84	9,2	**2.06 (1.59-2.67)**	**2.05 (1.57-2.66)**	61	7,2	**1.54 (1.14-2.06)**	**1.49 (1.10-2.00)**
Comparators	256	4,8	Ref.	Ref.	306	5,2	Ref.	Ref.	339	5,8	Ref.	Ref.

**Adjusted for country of birth and level of education.*
